# Medical Specialty Recommendations by an Artificial Intelligence Chatbot on a Smartphone: Development and Deployment

**DOI:** 10.2196/27460

**Published:** 2021-05-06

**Authors:** Hyeonhoon Lee, Jaehyun Kang, Jonghyeon Yeo

**Affiliations:** 1 Department of Clinical Korean Medicine Graduate School Kyung Hee University Seoul Republic of Korea; 2 Department of Computer Science Yonsei University Seoul Republic of Korea; 3 School of Computer Science and Engineering Pusan National University Busan Republic of Korea

**Keywords:** artificial intelligence, chatbot, COVID-19, deep learning, deployment, development, machine learning, medical specialty, natural language processing, recommendation, smartphone

## Abstract

**Background:**

The COVID-19 pandemic has limited daily activities and even contact between patients and primary care providers. This makes it more difficult to provide adequate primary care services, which include connecting patients to an appropriate medical specialist. A smartphone-compatible artificial intelligence (AI) chatbot that classifies patients’ symptoms and recommends the appropriate medical specialty could provide a valuable solution.

**Objective:**

In order to establish a contactless method of recommending the appropriate medical specialty, this study aimed to construct a deep learning–based natural language processing (NLP) pipeline and to develop an AI chatbot that can be used on a smartphone.

**Methods:**

We collected 118,008 sentences containing information on symptoms with labels (medical specialty), conducted data cleansing, and finally constructed a pipeline of 51,134 sentences for this study. Several deep learning models, including 4 different long short-term memory (LSTM) models with or without attention and with or without a pretrained FastText embedding layer, as well as bidirectional encoder representations from transformers for NLP, were trained and validated using a randomly selected test data set. The performance of the models was evaluated on the basis of the precision, recall, F_1_-score, and area under the receiver operating characteristic curve (AUC). An AI chatbot was also designed to make it easy for patients to use this specialty recommendation system. We used an open-source framework called “Alpha” to develop our AI chatbot. This takes the form of a web-based app with a frontend chat interface capable of conversing in text and a backend cloud-based server application to handle data collection, process the data with a deep learning model, and offer the medical specialty recommendation in a responsive web that is compatible with both desktops and smartphones.

**Results:**

The bidirectional encoder representations from transformers model yielded the best performance, with an AUC of 0.964 and F_1_-score of 0.768, followed by LSTM model with embedding vectors, with an AUC of 0.965 and F_1_-score of 0.739. Considering the limitations of computing resources and the wide availability of smartphones, the LSTM model with embedding vectors trained on our data set was adopted for our AI chatbot service. We also deployed an Alpha version of the AI chatbot to be executed on both desktops and smartphones.

**Conclusions:**

With the increasing need for telemedicine during the current COVID-19 pandemic, an AI chatbot with a deep learning–based NLP model that can recommend a medical specialty to patients through their smartphones would be exceedingly useful. This chatbot allows patients to identify the proper medical specialist in a rapid and contactless manner, based on their symptoms, thus potentially supporting both patients and primary care providers.

## Introduction

The COVID-19 pandemic has encouraged the development of telemedicine and the use of digital platforms [1]. In the field of remote medical support, various digital tools help minimize the number of face-to-face interactions between patients and health care providers (HCPs) [2]. Artificial intelligence (AI) chatbots, also called conversational agents, have recently been designed to support HCPs [3]. Most AI chatbots utilize deep learning–based natural language processing (NLP), which can analyze natural human language input and respond appropriately in a conversational manner [4]. The advantages of an AI chatbot over human HCPs include the absence of face-to-face interaction; minimization of bias based on certain patient demographic characteristics such as age, gender, and race; greater cost-effectiveness; and 24/7 availability since the chatbot does not get fatigued or sick [5]. In a recent systematic review on the effectiveness of AI chatbots in health care, the bots performed well in terms of both usability and satisfaction, and overall positive or mixed effectiveness was reported in most studies [6].

In primary care, it may be important for HCPs to determine which medical specialty is most appropriate for their patients. Since patients generally have no professional medical knowledge, they have to rely on the decisions made by the primary care provider. It cannot be overemphasized that high-quality primary care systems ensure favorable health outcomes and decrease the economic burden [7]. Currently, however, the COVID-19 pandemic limits the amount of physical contact between patients and primary care providers. This state of affairs hinders communication between patients and primary care providers, which prevents timely provision of appropriate treatment by a medical specialist and worsens health outcomes. Therefore, the need for digital tools including AI chatbots to complement the care provided by HCPs and support the decision-making capacity of primary care providers (for example, by connecting patients to medical specialists) is greater.

To develop AI chatbots, electronic medical records (EMRs) have generally been used as input data pipelines for NLP-related medical studies. Using EMRs for NLP facilitates the identification of patients with digestive disorders [8,9] and the prediction of the risk of psychiatric problems, such as actual self-harm, harm to others or victimization, and the risk of health care–associated infections such as surgical site infections [10,11]. Furthermore, EMRs have been used to develop an excellent medical specialty classifier built using deep learning–based NLP, which reportedly had area under receiver operating characteristic curve (AUC) scores of 0.975 and 0.991 and F_1_-scores of 0.845 and 0.870 in 2 different EMR data sets [12]. However, the text-formatted EMR data generated by HCPs are mainly composed of medical terminology, which differs from the expressions used by patients to describe their symptoms. Therefore, a new data set consisting of sentences that are commonly used by patients to ask their HCPs about their symptoms was required to fulfill the purpose of our chatbot. This study aims to collect data that accurately describes patients’ symptoms in a real-world setting (much more friendly to patients than HCPs) and to develop a deep learning–based NLP model for medical specialty classification. Specifically, we constructed various deep learning–based NLP models, compared their performances, and then selected the best model for our AI chatbot. Finally, the developed AI chatbot was deployed in Google Cloud, which can be used on both desktops and smartphones.

## Methods

### Data Collection and Cleansing

For supervised learning of the deep learning–based model for NLP, both a single-sentence symptom description and its corresponding medical specialty were required. A Korean website called HiDoc [13]—a web-based health care platform—provides a medical consultation service for anonymous users (patients) by linking them to more than 4000 medical specialists. All medical specialists submitted their professional licenses for approval to provide medical consultation to HiDoc users. HiDoc posts, in which users describe their symptoms, have two parts: title and content. The titles of the posts, in a single-sentence format, were collected for our data set. The medical specialty corresponding to each title sentence was obtained from the profile of the medical specialist who responded to the post.

In the first step of the data cleansing process, duplicate and missing data were eliminated. Second, ambiguous sentences that were not sufficient for accurate classification of the medical specialty, including sentences with ≤2 words or those not related to medical consultations, were manually excluded. Third, very few instances of mislabeled data were appropriately relabeled by a well-trained physician.

### Exploratory Data Analysis

Exploratory data analysis (EDA) was performed to extract the interpretable features of the data before development of the deep learning–based NLP models. First, we enumerated the sentences related to symptoms in each class to assess the data distribution. We also visualized the most frequently used words to create lists of words, such as stop words (not useful for classification) and keywords (useful for classification) for word representation. The sentence lengths were determined (in terms of both word count and character count) to ascertain the maximum length of the input sequence for each model.

### Development of Deep Learning Models

#### Long Short-Term Memory Models

Clinical word representation has proven to be an important factor in the performance of NLP models [14]. To extract the appropriate word representation, each sentence was mined for nouns excluding the words in the list of stop words, and each noun was converted to an index if included in the list of keywords. Using a tokenizer in the Keras library, 15,000 high-frequency nouns were replaced with the corresponding numbers. Thereafter, padding tokens were added to the sentence to ensure consistency in sentence length. As the input of the long short-term memory (LSTM) models, we used either word embedding vectors trained on our data set or word embedding vectors pretrained with the Korean corpus from FastText. The number of embedding dimensions was set to 2048. As the fundamental LSTM architecture, a 256-cell bidirectional LSTM served as the backbone of the model with or without an added attention layer [15,16]. Further, 2 fully connected layers with a rectified linear unit function were applied, followed by a dense layer with the softmax function for classification.

We built 4 different LSTM models. The first is the LSTM model with embedding vectors trained on our data set. Second is the LSTM model with Bahdanau attention. Most settings are the same in the first and second models, but the second model includes an attention layer with 256 cells after the bidirectional LSTM layer. The third model is the LSTM model with FastText pretrained vectors. We loaded FastText's pretrained vector data set and reorganized it to create the embedding matrix. This matrix was used as the embedding layer's weight. The unmentioned hyperparameters are the same as those in the first LSTM model. The last variation is the LSTM model with both FastText vectors and Bahdanau attention. It has an embedding layer based on FastText pretrained vectors and a Bahdanau attention layer.

When compiling all 4 models, categorical cross-entropy and Adam were applied as the loss function and training optimizer, respectively. For the training process, 10-fold cross-validation was conducted to ensure evaluation accuracy. Furthermore, early stopping callback monitoring validation loss was used to prevent overfitting. The batch size and number of epochs were set to 1000 and 30, respectively.

#### Bidirectional Encoder Representations From Transformers Model

The bidirectional encoder representations from transformers (BERT) model, proposed by Google, has achieved state-of-the-art performance on biomedical and clinical entity normalization with EMRs as well as other NLP tasks such as question answering and natural language inference [17]. Therefore, we used the BERT model for sentence classification through fine-tuning. For preprocessing of the sentences, we applied an open-source tokenizer from Huggingface [18]. This tokenizer encoded each sentence to be used as input of the BERT model by, for example, adding special tokens ([CLS] for beginning and [SEP] for end of sentence), padding to the maximum sequence length, and generating an attention mask. The BERT classification model was built from the pretrained BERT model with a fully connected layer and the softmax function on top. Categorical cross-entropy and Adam optimizer were used for compilation. As in the LSTM models, 10-fold cross-validation was used to increase the reliability of the statistical findings, and early stopping callback observing “validation loss” with patience 2 was set to avoid overfitting. Training was carried out for 30 epochs per fold, with a batch size of 100.

### Evaluation

We performed 10-fold cross-validation for the entire data set for each model, and then we calculated the mean score of the 10 different folds to evaluate the general performance of the models. Model performance was evaluated on the basis of precision, recall, F_1_-score, and AUC. These indexes are calculated from the rates of true positive, false positive, and false negative results, as follows:



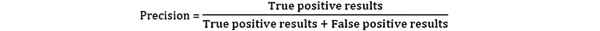





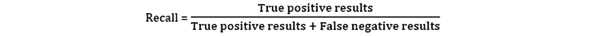





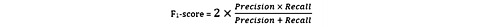



AUC = area under the curve of the false positive rate (x-axis) vs the true positive rate (y-axis).

The Wilcoxon signed rank test was used to test the significance of between-group differences.

### Development of the AI Chatbot

We next developed a user-friendly application that allows patients to interact with our chatbot model. As we aimed to make our chatbot usable for all, without creating any digital health disparities on the basis of factors including age, and ensure that it provided accurate medical information, we adopted the formal tone of the Korean language. A well-designed chatbot provides agility for developers and is able to run continuously in any environment. We constructed a chatbot architecture considering those factors.

A typical chatbot architecture can be simplified into 2 parts. The first part is the client-side showing the main user interface. The other part is the server-side that includes the dialog processing logic and an NLP model.

We developed a prototype chatbot client using an open-source chatbot framework Alpha [19]. There are various chatbot user interface alternatives to Alpha, including “chat-bubble,” but Alpha is superior for several reasons. In general, open-source chatbot user interface frameworks only have features for sending and receiving messages. They are difficult to test or execute in a developer environment. The Alpha chatbot framework is a highly customizable, fully complete chatbot framework. Alpha is predockerized and built with a WebKit, which may help developers quickly run and test the continuously changing codebase. Furthermore, Alpha includes a cross-platform feature that allows it to be used on both desktops and smartphones. For rapid development, we modified Alpha's client-side dialog logic to fit with the targeted user base.

### Tools

Frequently used words were visualized in a word cloud using a Python package called “WordCloud.” The Python package for Korean NLP “KoNLPy” was used for word representation. The Huggingface “Transformers” package was used to encode sentences and load a pretrained model for BERT. The “Tensorflow” framework was adopted for building and evaluating the deep learning models. Google Colab, a cloud service for machine learning research, was used in this study. It provides various libraries and frameworks for deep learning and a robust graphics processing unit. Statistical analysis was conducted using R (version 4.0.3, The R Foundation).

## Results

### Data Set Construction

**Figure 1:**
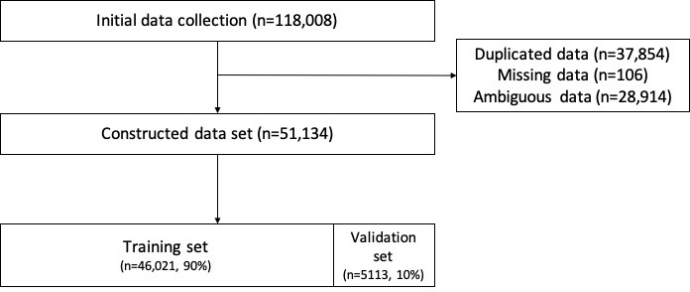
Flow diagram for data set construction.

We initially collected 118,008 sentences that discussed patients’ symptoms, which fit into the 26 classes of medical specialty in HiDoc, and eliminated duplicate data (n=37,854) and missing data (n=106). After excluding ambiguous sentences (n=28,914), the final data set including 51,134 sentences in 26 medical specialty classes was constructed. The data set was randomly split into a training set (n=66,021, 90%) and validation set (n=5,113, 10%) during 10-fold cross-validation. The flow diagram of the entire data set construction process is shown in Figure 1.

### EDA

By specialty, the number of sentences was the highest in dermatology (19.87%), followed by psychology (12.04%), neurology (9.93%), and orthopedic surgery (7.10%) (Table 1).

As the word cloud showed that some frequent words including daily expressions were predominant in the data set, we investigated the list of frequent words. Furthermore, some useful statistical information could be identified at the word and character levels (Table 2).

The EDA results provided 3 crucial ideas to help identify the best NLP model for the AI chatbot. First, owing to the class imbalance of the data, the F_1_-score should be considered the most important measure to accurately evaluate and compare the models. Second, to improve the performance of the models through data preprocessing and tokenizing, we decided to compile a list of stop words, such as “hello,” “question,” and “ask,” which might not be helpful for classification, and a list of keywords, which are frequently found in medical expressions (n=15,000), such as “pain,” “head,” and “sudden.” Third, considering the sentence length at both levels, the maximum length of the input sequences for each deep learning model (10 for the LSTM models and 30 for the BERT model) was determined to fix the shape of the input layer in each model.

**Table 1:** Number of sentences describing symptoms in 26 medical specialty classes.

Medical specialty	Sentences, n (%)
Dermatology	10172 (19.89)
Psychology	6154 (12.04)
Neurology	5080 (9.93)
Orthopedic surgery	3628 (7.10)
Gastroenterology	3096 (6.05)
Otorhinolaryngology	3065 (5.99)
Ophthalmology	2852 (5.58)
Neurosurgery	2028 (3.97)
Rehabilitation medicine	1934 (3.78)
Cardiology	1640 (3.21)
Pulmonology	1334 (2.61)
Plastic surgery	1292 (2.53)
Korean traditional medicine	1227 (2.40)
Obstetrics and gynecology	1170 (2.29)
Infectious disease	1115 (2.18)
Dentistry	1100 (2.15)
Endocrinology	970 (1.90)
Cardiothoracic surgery	713 (1.39)
Rheumatology	654 (1.28)
Urology	521 (1.02)
Anesthesiology	418 (0.82)
Nephrology	283 (0.55)
Hematology and oncology	273 (0.53)
Allergy and immunology	227 (0.44)
General surgery	117 (0.23)
Emergency medicine	71 (0.14)

**Table 2:** Features of sentence length at the word and character level.

Statistic	Word level, n	Character level, n
Maximum	52	156
Minimum	1	1
Mean	4.68	20.14
Median	4	18
SD	2.78	11.03
First quartile	3	12
Third quartile	6	27

### Comparison of Deep Learning Models

The performance outcomes after 10-fold cross-validation in the 5 different deep learning models for NLP are summarized in Table 3. The BERT model showed the best performance, followed by the LSTM model, with embedding vectors trained on our data set.

After saving all trained models in the server, we determined that the BERT model was too heavy to be run on our GCP computation engine with limited performance because the server should be able to handle requests on both desktops and smartphones. Therefore, we had to utilize the lighter LSTM model with embedding vectors trained on our data set, which showed the second-best classification performance.

**Table 3:** Comparison of classification performance among the 5 different deep learning models during 10-fold cross-validation.

Model#	Word embedding	Model	Precision (95% CI)	Recall (95% CI)	F_1_-score (95% CI)	Area under the receiver operating characteristic curve (95% CI)	*P* value
1	Trained on our own data set	Long short-term memory	0.805 (0.800-0.810)	0.686 (0.684-0.689)	0.739 (0.737-0.742)	0.965 (0.964-0.966)	<.01
2	Trained on our own data set	Long short-term memory+attention	0.798 (0.794-0.801)	0.672 (0.668-0.675)	0.727 (0.725-0.730)	0.959 (0.957-0.960)	<.01
3	Pretrained from FastText	Long short-term memory	0.789 (0.786-0.791)	0.622 (0.617-0.627)	0.693 (0.689-0.696)	0.963 (0.962-0.964)	Reference^a^
4	Pretrained from FastText	Long short-term memory+attention	0.800 (0.796-0.803)	0.645 (0.638-0.651)	0.711 (0.707-0.716)	0.965 (0.964-0.966)	<.01
5	Pretrained from Transformers	Bidirectional encoder representations from transformers	0.799 (0.795-0.803)	0.740 (0.737-0.743)	0768 (0.766-0.769)	0.964 (0.963-0.965)	<.01

^a^Comparison of F_1_-scores of the models with the reference, which showed the lowest F_1_-score.

### Deployed AI Chatbot

**Figure 2:**
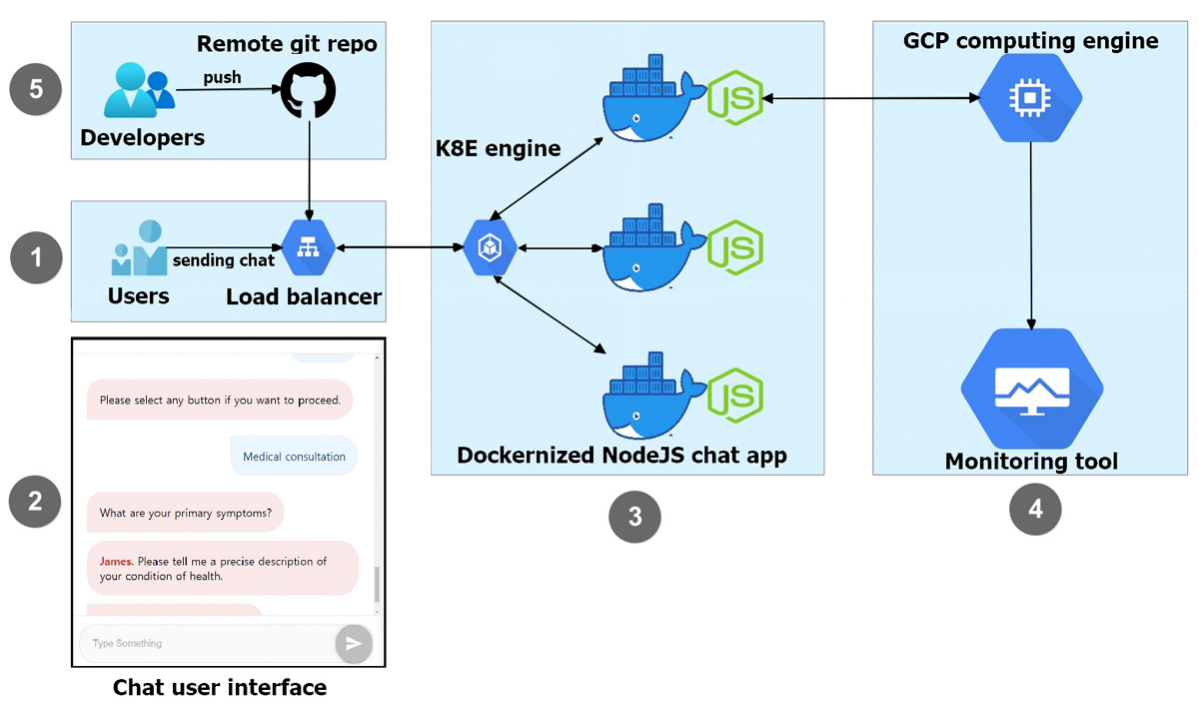
Architecture of the chatbot. This figure illustrates the workflow of the developed prototype chatbot. (1) Users send a sentence through the chatbot's input box. (2) A simple example of our chatbot’s user interface. (3) The Containerized Node js chat application includes responding logic without classifying a medical department. (4) The GCP computing engine, which has a natural language processing model, which classifies the medical department from the sentence input in (3). (5) This dockerized NodeJS-based chat app is deployed by continuous git push steps.

Figure 2 presents the overall architecture of our chatbot. On top of the complete chatbot features, we added basic bubble-like buttons that classify medical departments using our deep learning model, link to an online appointment system, list medical doctors, and provide a simple introduction of the chatbot. When a user selects “medical consultation,” the chatbot asks to send a natural Korean language sentence that describes the user's health state. The sentence is sent to a deployed server and the NLP model classifies medical specialties using the given information. The server responds to the client with the classified medical specialty. The user's device shows the complete sentence generated on the client-side. Based on the medical specialty outcome, the chatbot asks the user if it should provide some services, such as presenting the schedule of physicians in the medical specialty and helping to make an appointment with a physician.

Our NLP model was deployed to a server-side e2-medium (2 vCPU, 4 GB memory) GCP computation engine. We deployed this chatbot framework on Google Cloud Platform Kubernetes Engine (GKE), which is conveniently coupled with containerized by docker, for rapid development. GKE's Kubernetes has a load-balancer feature that distributes application traffic to prevent sudden chatbot outages. Applying Kubernetes at an early stage of development helps developers optimize the uptime, performance, and cost of the application.

We also applied automated git automation with Kubernetes to accelerate the speed of delivery. When a developer pushes modified code from a local computer to a remote GitHub repository, GitHub triggers the containerized chat app cloned in GKE. This continuous deployment method eases the burden on developers by eliminating the need for complex configured scripts on a deployment server. In the cloud-native environment, this chatbot framework laid the groundwork to easily extend further features.

## Discussion

### Principal Findings

In this study, the BERT model was the best classifier of medical specialty, followed by the LSTM model with embedding vectors trained on our data set. These 2 models had an AUC of 0.964 and 0.965 and F_1_-score of 0.768 and 0.739, respectively; however, these values were lesser than those reported previously (AUC of 0.975-0.991 and F_1_-score of 0.845-0.870) [12]. Nonetheless, the main findings of this study include the following: (1) we developed not only a deep learning classification model for medical sentences but also a prototype AI chatbot to be executed on a smartphone; (2) to our knowledge, this study is the first to use real patients’ actual descriptions of their symptoms to develop a deep learning–based NLP model for medical specialty classification; and (3) we constructed Korean language data sets, which can be used in further studies.

This AI chatbot may help patients understand which medical specialty is appropriate for the treatment of their present symptoms and then make an appointment with the corresponding medical specialist, without any face-to-face contact throughout the process. Previous deep learning–based studies on medical sentences developed and suggested optimized deep learning models for several purposes [12,20-23]. However, even with these deep learning models, it takes a great deal of time, effort, and trial and error to deploy a web-based service for practical use. We also made the final choice of deep learning model considering performance and size before deployment. Similar to various recently developed web-based medical tools that offer assessment, communication, management, and other features [24-28], we also focused on the accessibility of our AI chatbot among users with smartphones. As the use of AI techniques in medical services such as telemedicine is growing owing to the COVID-19 pandemic, we believe that this type of research (from the development of a deep learning model to deployment of an AI chatbot) has become extremely important as it can be rapidly applied in practical medical settings.

Most NLP-related medical studies have used EMRs such as clinical notes and discharge notes [21,29,30]. These types of medical texts consist of “physician-friendly” words; that is, medical terminology. Therefore, in those studies, patients’ expressions of their own complaints must be transformed in an appropriate manner before being used as input for the model. It was also not suitable for the input of the AI chatbot. However, the data set constructed and used for the development of the deep learning–based NLP model in this study consists of “patient-friendly” words taken from a currently operating web-based health care platform. The use of this data set helped develop an AI chatbot with which patients can interact easily and conveniently. Furthermore, this data set of Korean medical language may be useful for further NLP-based medical studies, including those on diagnosis, treatment, and prediction [31-33] because different languages have different features that can greatly influence the study of NLP.

### Limitations

One limitation of this study is that the corpus of the data set that was used for the development of the NLP models was obtained from one specific website. Demographically, most HiDoc users who accessed the website were less than 65 years of age (Multimedia Appendix 1). They also have nonemergent symptoms that are not complex enough to be differentially diagnosed and not too sensitive to readily share with others. This may explain why the largest proportion of symptom-related sentences were associated with dermatology, followed by psychology (Table 1). Second, the present chatbot, which uses text messaging for remote communication, might be uncomfortable for older adults. To not ignore the needs of this vulnerable and continuously increasing population, an additional communication strategy, such as voice-based communication, is required. A recent study reported that a smart speaker was an effective solution for bringing an AI-based digital health system for older Korean adults [34]. Therefore, other AI-based technologies including smart speakers should be considered for further studies. Third, our model provides classification into 26 medical specialties based on the system of a single general hospital. This may affect the generalizability of our model; a few medical specialties that exist in other medical facilities may be missing. Fourth, some aspects of the study may have been restricted, such as the amount or quality of the data and computing resources. To improve the performance of the deep learning model for classification, additional large collections of high-quality data as well as a costly, latest-generation high-performance computation engine could be used for a large model such as BERT. Fifth, concerning the interpretability of deep learning models, the use of shallow learning models such as support vector machines and naïve Bayes classifiers might be suggested for further studies. Sixth, a clinical trial is required to evaluate how well our AI chatbot service chooses the correct medical specialty in a real-world setting.

### Conclusions

In this study, we illustrate the potential of a smartphone-compatible AI chatbot service to recommend a suitable medical specialty to patients. We developed a deep learning–based NLP model and deployed the novel AI chatbot. This type of non–face-to-face medical service is a promising strategy to overcome the current difficulties associated with the COVID-19 pandemic.

## Appendix

Multimedia Appendix 1 Demographic data of HiDoc users.

## Abbreviations

AI: artificial intelligence
AUC: area under receiver operating characteristic curve
BERT: bidirectional encoder representations from transformers
EDA: exploratory data analysis
EMR: electronic medical record
GKE: Google Cloud Platform Kubernetes Engine
HCP: health care provider
LSTM: long short-term memory
NLP: natural language processing
