# Supplementary material for: Medical Specialty Recommendations by an Artificial Intelligence Chatbot on a Smartphone: Development and Deployment
Source: J Med Internet Res. 2021 May 6;23(5):e27460. doi: 10.2196/27460 (PMC8104000; doi:10.2196/27460)
Supplement: Multimedia Appendix 1 [file jmir_v23i5e27460_app1.docx]

Multimedia Appendix 1. Demographic data of HiDoc users.

|  |  | Proportion (%) |
| --- | --- | --- |
| Age | 65+ | 7.12 |
|  | 55-64 | 19.11 |
|  | 45-54 | 30.87 |
|  | 35-44 | 20.13 |
|  | 25-34 | 15.39 |
|  | 18-24 | 7.38 |
| Sex | Female | 55.95 |
|  | Male | 44.05 |
